# Supplementary material for: Ratchet-free solid-state inertial rotation of a guest ball in a tight tubular host
Source: Nat Commun. 2018 May 15;9:1907. doi: 10.1038/s41467-018-04325-2 (PMC5954156; doi:10.1038/s41467-018-04325-2)
Supplement: Supplementary file 1 — Supplementary Information [file 41467_2018_4325_MOESM1_ESM.pdf]

Supplementary Information

Ratchet-free solid-state inertial rotation of a guest ball in a tight tubular host

Taisuke Matsuno, Yusuke Nakai, Sota Sato, Yutaka Maniwa & Hiroyuki Isobe

Supplementary Figures

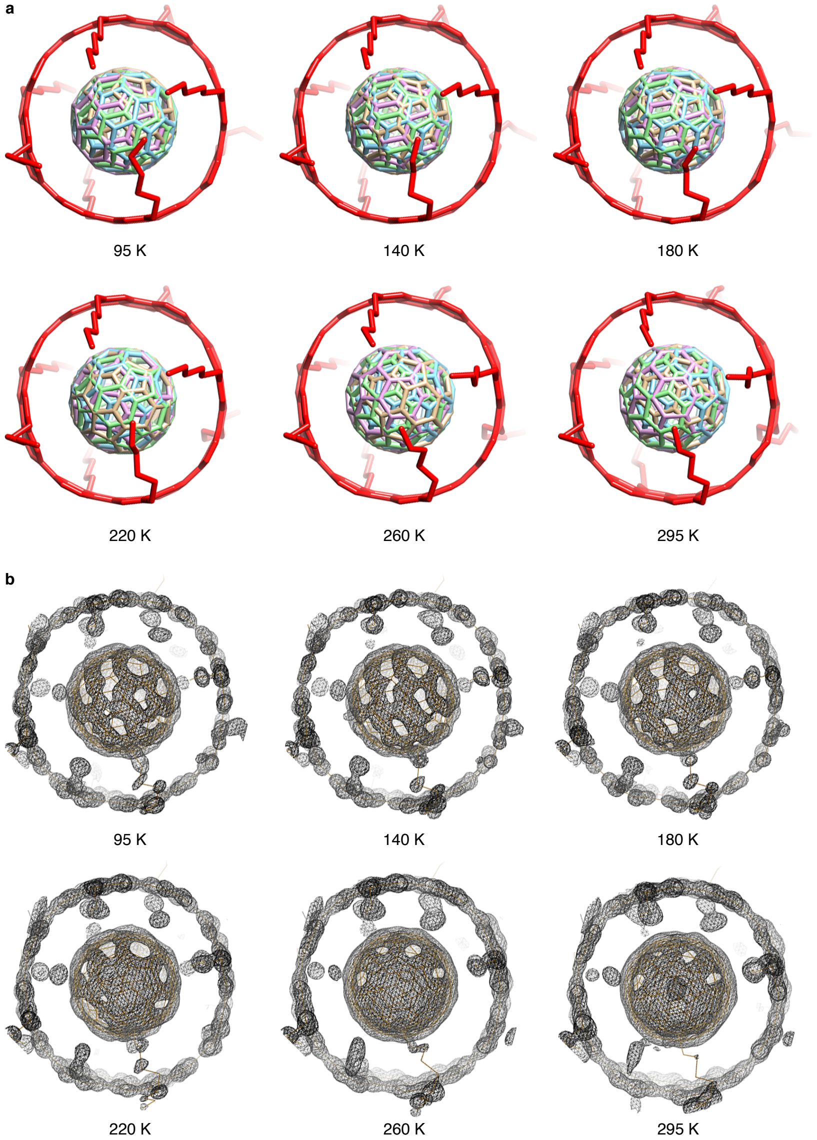

Supplementary Fig. 1 Crystallographic analyses of (*P*)-(12,8)-[4]CC⊃C_{60} at 95, 140, 180, 220, 260 and 295 K. **a**, Crystal structures shown in tube models. Four different orientations of C_{60} were shown in different colors. Disordered alkyl chains and hydrogen atoms are omitted for clarity. **b**, Electron density mappings with $2F_o$–F_c (RMSD: 1.5σ).

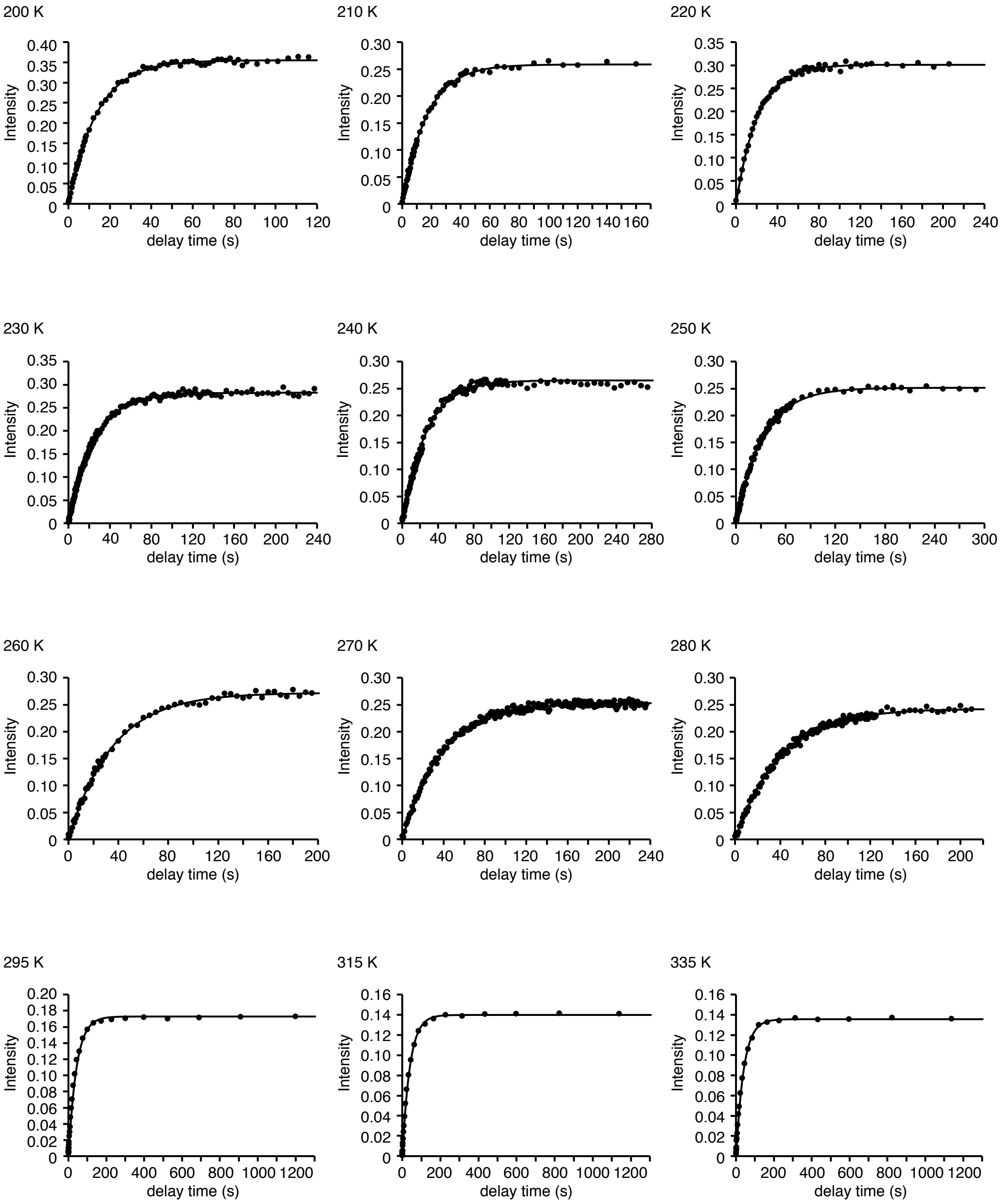

Supplementary Fig. 2 Raw data of VT T_1 measurements with (*P*)-(12,8)-[4]CC$\supset$C$_{60}$ under 4.01 T.

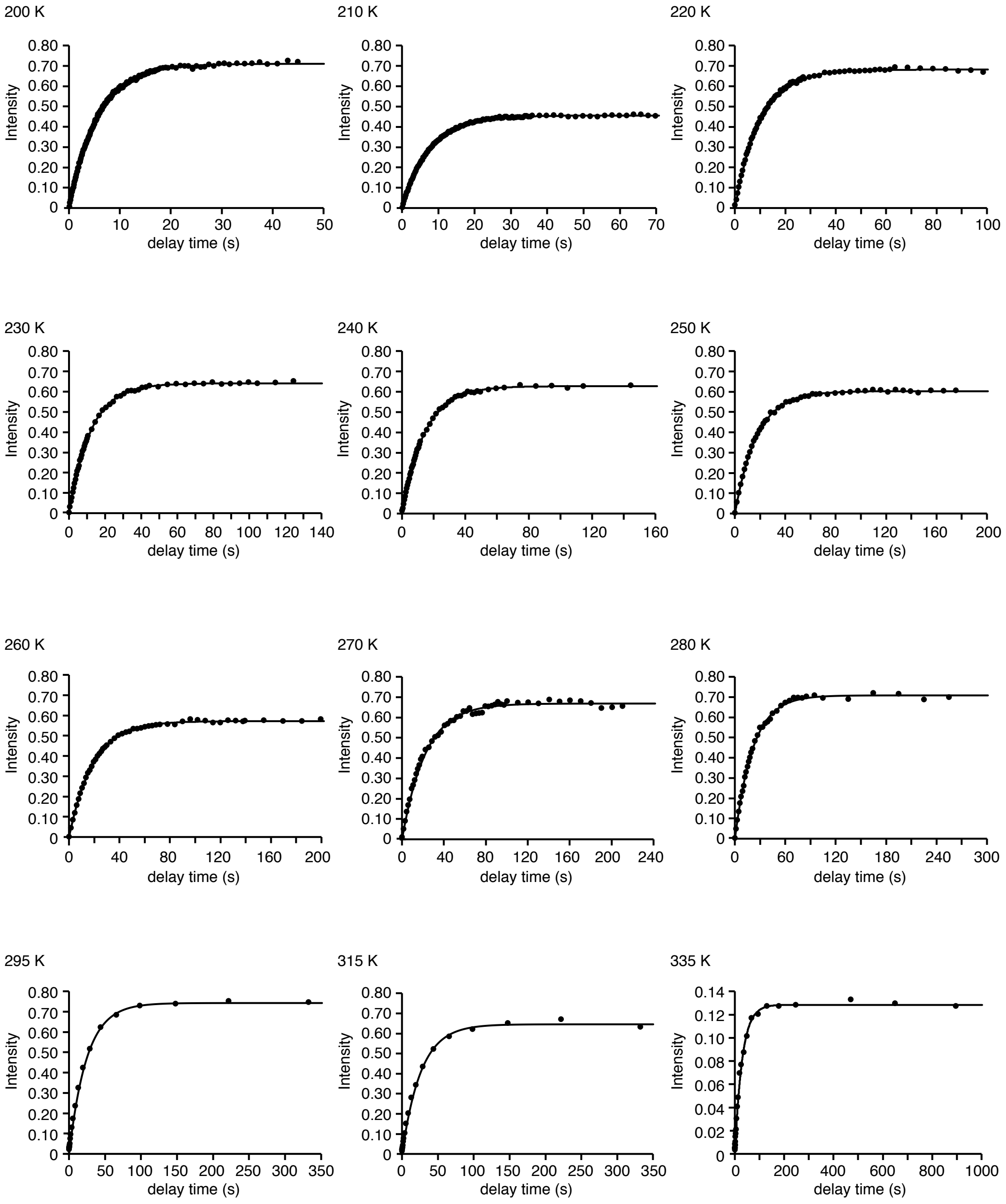

Supplementary Fig. 3 Raw data of VT T_1 measurements with (P)-(12,8)-[4]CC$\supset$$C_{60}$ under 9.40 T.

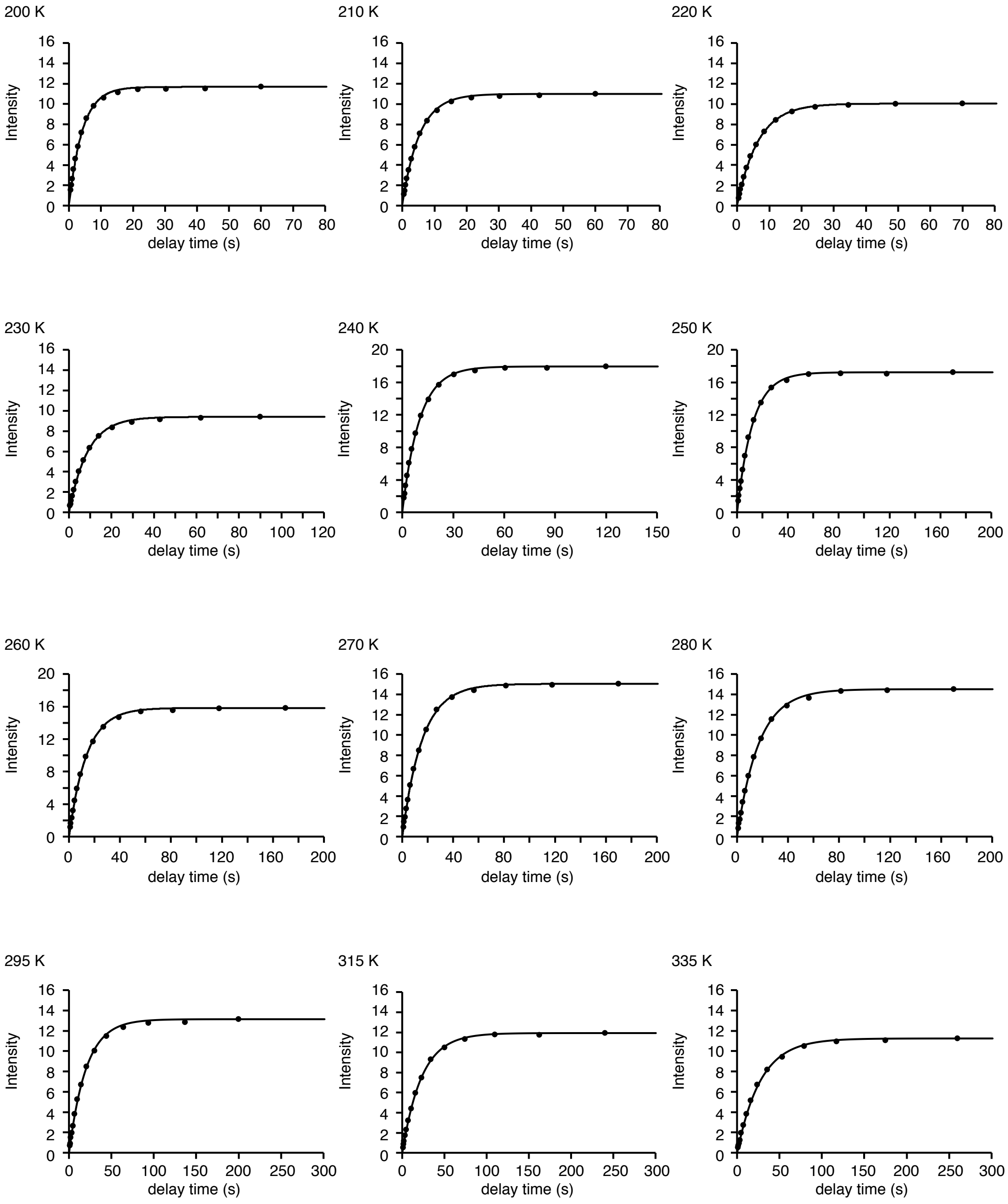

Supplementary Fig. 4 Raw data of VT T_1 measurements with (*P*)-(12,8)-[4]CC$\supset C_{60}$ under 11.7 T.

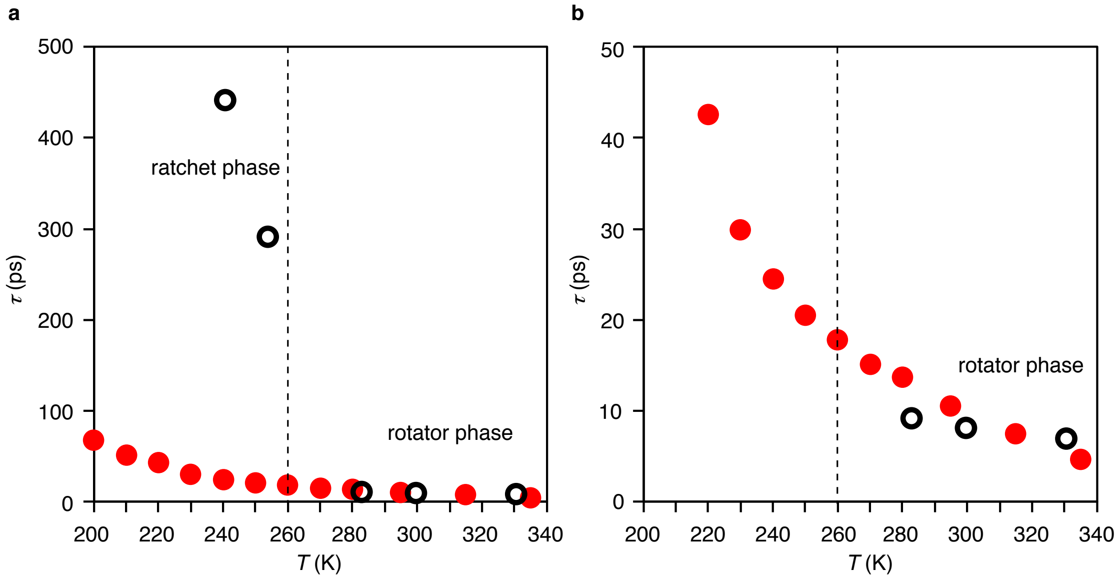

Supplementary Fig. 5 Comparisons of τ values of (*P*)-(12,8)-[4]CC$\supset C_{60}$ with those of intact C_{60}. The data for the intact C_{60} was taken from the literature[2]. The data of (*P*)-(12,8)-[4]CC$\supset C_{60}$ are shown with red filled circles, and the data of C_{60} are shown in open black circles. **a**, Whole-range data to show the presence of the phase transition (ratchet/rotator) with C_{60}. **b**, A close-up view to show the absence of phase transition (ratchet/rotator) with (*P*)-(12,8)-[4]CC$\supset C_{60}$.

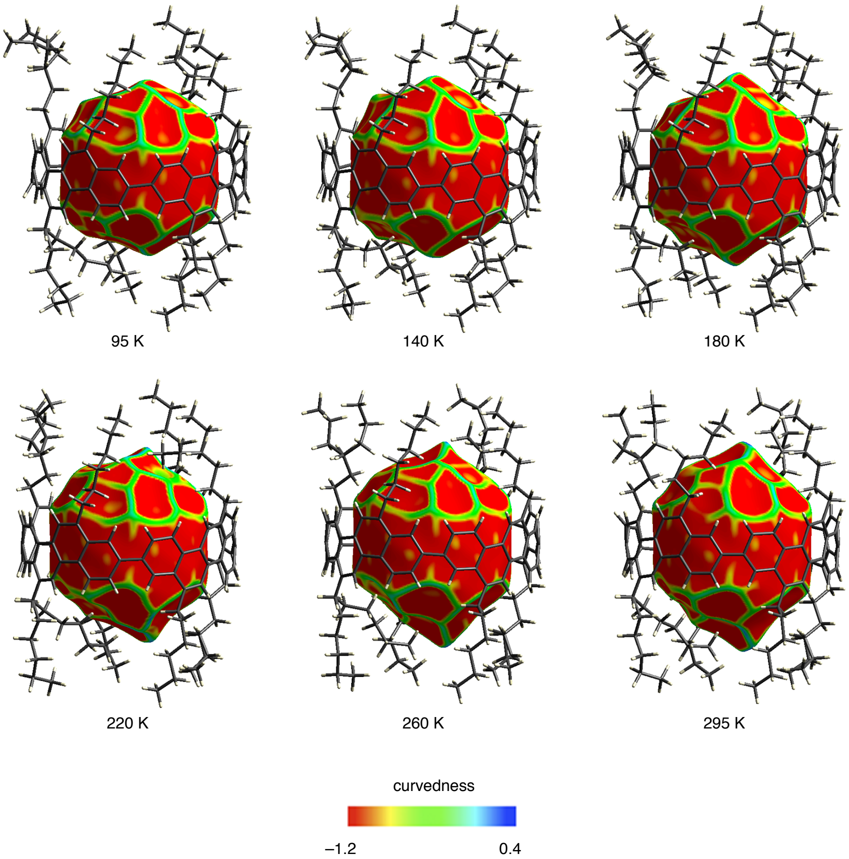

Supplementary Fig. 6 Hirshfeld surfaces of C_{60} in (*P*)-(12,8)-[4]CC mapped with curvedness. No inflection lines were observed (see also ref. 15).

Supplementary Tables

Supplementary Table 1 Crystal data and structure refinement at 95 K (CCDC 1821725)

Empirical formula	$C_{182.50}$ H_{141} Cl_5	
Formula weight	2511.19	
Temperature	95(2) K	
Wavelength	0.80000 Å	
Crystal system	Trigonal	
Space group	$P\,3_1$	
Unit cell dimensions	a = 28.980(4) Å	α = 90°.
	b = 28.980(4) Å	β = 90°.
	c = 13.230(3) Å	γ = 120°.
Volume	9622(3) $Å^3$	
Z	3	
Density (calculated)	1.300 Mg/m^3	
Absorption coefficient	0.233 mm^{-1}	
F(000)	3963	
Crystal size	0.100 × 0.100 × 0.050 mm^3	
Theta range for data collection	1.582 to 28.840°.	
Index ranges	–34<=h<=34, –34<=k<=34, –15<=l<=15	
Reflections collected	113344	
Independent reflections	23472 [R(int) = 0.0403]	
Completeness to theta = 28.685°	100.0 %	
Absorption correction	Semi-empirical from equivalents	
Max. and min. transmission	1.000 and 0.686	
Refinement method	Full-matrix least-squares on F^2	
Data / restraints / parameters	23472 / 3225 / 2770	
Goodness-of-fit on F^2	2.396	
Final R indices [I>2sigma(I)]	R_1 = 0.1213, wR_2 = 0.2931	
R indices (all data)	R_1 = 0.1397, wR_2 = 0.3081	
Absolute structure parameter	0.024(13)	
Extinction coefficient	n/a	
Largest diff. peak and hole	0.370 and –0.461 e.$Å^{-3}$	

Supplementary Table 2 Crystal data and structure refinement at 140 K (CCDC 1821727)

Empirical formula	$C_{182.50} H_{141} Cl_5$	
Formula weight	2511.19	
Temperature	140(2) K	
Wavelength	0.80000 Å	
Crystal system	Trigonal	
Space group	$P\,3_1$	
Unit cell dimensions	a = 29.080(4) Å	α = 90°.
	b = 29.080(4) Å	β = 90°.
	c = 13.330(3) Å	γ = 120°.
Volume	9762(3) Å^3	
Z	3	
Density (calculated)	1.281 Mg/m^3	
Absorption coefficient	0.230 mm^{-1}	
F(000)	3963	
Crystal size	0.200 × 0.100 × 0.100 mm^3	
Theta range for data collection	1.576 to 28.826°.	
Index ranges	$-34<=h<=35$, $-35<=k<=35$, $-16<=l<=16$	
Reflections collected	111900	
Independent reflections	23817 [R(int) = 0.0403]	
Completeness to theta = 28.685°	100.0 %	
Absorption correction	Semi-empirical from equivalents	
Max. and min. transmission	1.000 and 0.512	
Refinement method	Full-matrix least-squares on F^2	
Data / restraints / parameters	23817 / 3231 / 2770	
Goodness-of-fit on F^2	2.924	
Final R indices [I>2sigma(I)]	R_1 = 0.1394, wR_2 = 0.3425	
R indices (all data)	R_1 = 0.1505, wR_2 = 0.3524	
Absolute structure parameter	0.020(13)	
Extinction coefficient	n/a	
Largest diff. peak and hole	0.510 and –0.523 e.Å^{-3}	

Supplementary Table 3 Crystal data and structure refinement at 180 K (CCDC 1821730)

Empirical formula	$C_{182.50}$ H_{141} Cl_5	
Formula weight	2511.19	
Temperature	180(2) K	
Wavelength	0.80000 Å	
Crystal system	Trigonal	
Space group	$P\,3_1$	
Unit cell dimensions	a = 29.130(4) Å	α = 90°.
	b = 29.130(4) Å	β = 90°.
	c = 13.410(3) Å	γ = 120°.
Volume	9855(3) $Å^3$	
Z	3	
Density (calculated)	1.269 Mg/m^3	
Absorption coefficient	0.227 mm^{-1}	
F(000)	3963	
Crystal size	0.200 × 0.200 × 0.100 mm^3	
Theta range for data collection	1.574 to 28.825°.	
Index ranges	–35<=h<=35, –35<=k<=35, –16<=l<=16	
Reflections collected	111365	
Independent reflections	23996 [R(int) = 0.0436]	
Completeness to theta = 28.685°	100.0 %	
Absorption correction	Semi-empirical from equivalents	
Max. and min. transmission	1.000 and 0.447	
Refinement method	Full-matrix least-squares on F^2	
Data / restraints / parameters	23996 / 3239 / 2770	
Goodness-of-fit on F^2	2.927	
Final R indices [I>2sigma(I)]	R_1 = 0.1396, wR_2 = 0.3455	
R indices (all data)	R_1 = 0.1487, wR_2 = 0.3535	
Absolute structure parameter	0.028(17)	
Extinction coefficient	n/a	
Largest diff. peak and hole	0.491 and –0.512 e.$Å^{-3}$	

Supplementary Table 4 Crystal data and structure refinement at 220 K (CCDC 1821728)

Empirical formula	$C_{182.50} H_{141} Cl_5$	
Formula weight	2511.19	
Temperature	220(2) K	
Wavelength	0.80000 Å	
Crystal system	Trigonal	
Space group	$P\,3_1$	
Unit cell dimensions	a = 29.240(4) Å	α = 90°.
	b = 29.240(4) Å	β = 90°.
	c = 13.500(3) Å	γ = 120°.
Volume	9996(3) $Å^3$	
Z	3	
Density (calculated)	1.252 Mg/m^3	
Absorption coefficient	0.224 mm^{-1}	
F(000)	3963	
Crystal size	0.200 × 0.100 × 0.100 mm^3	
Theta range for data collection	1.568 to 28.844°.	
Index ranges	$-35<=h<=35$, $-35<=k<=35$, $-16<=l<=16$	
Reflections collected	115877	
Independent reflections	24398 [R(int) = 0.0507]	
Completeness to theta = 28.685°	100.0 %	
Absorption correction	Semi-empirical from equivalents	
Max. and min. transmission	1.000 and 0.705	
Refinement method	Full-matrix least-squares on F^2	
Data / restraints / parameters	24398 / 3240 / 2770	
Goodness-of-fit on F^2	2.700	
Final R indices [I>2sigma(I)]	R_1 = 0.1428, wR_2 = 0.3322	
R indices (all data)	R_1 = 0.1535, wR_2 = 0.3391	
Absolute structure parameter	0.07(3)	
Extinction coefficient	n/a	
Largest diff. peak and hole	0.359 and –0.396 e.$Å^{-3}$	

Supplementary Table 5 Crystal data and structure refinement at 260 K (CCDC 1821726)

Empirical formula	$C_{182.50}$ H_{141} Cl_5	
Formula weight	2511.19	
Temperature	260(2) K	
Wavelength	0.80000 Å	
Crystal system	Trigonal	
Space group	$P\ 3_1$	
Unit cell dimensions	a = 29.320(4) Å	α = 90°.
	b = 29.320(4) Å	β = 90°.
	c = 13.540(3) Å	γ = 120°.
Volume	10080(3) $Å^3$	
Z	3	
Density (calculated)	1.241 Mg/m^3	
Absorption coefficient	0.222 mm^{-1}	
F(000)	3963	
Crystal size	0.200 × 0.200 × 0.100 mm3	
Theta range for data collection	0.903 to 28.851°.	
Index ranges	$-35<=h<=35$, $-35<=k<=35$, $-16<=l<=16$	
Reflections collected	77552	
Independent reflections	21210 [R(int) = 0.0365]	
Completeness to theta = 28.685°	99.1 %	
Absorption correction	Semi-empirical from equivalents	
Max. and min. transmission	1.000 and 0.818	
Refinement method	Full-matrix least-squares on F^2	
Data / restraints / parameters	21210 / 3203 / 2770	
Goodness-of-fit on F^2	1.145	
Final R indices [I>2sigma(I)]	R_1 = 0.1082, wR_2 = 0.2654	
R indices (all data)	R_1 = 0.1145, wR_2 = 0.2805	
Absolute structure parameter	0.09(5)	
Extinction coefficient	n/a	
Largest diff. peak and hole	0.220 and –0.217 e.$Å^{-3}$	

Supplementary Table 6 Crystal data and structure refinement at 295 K (CCDC 1821729)

Empirical formula	$C_{182.50}$ H_{141} Cl_5	
Formula weight	2511.19	
Temperature	295(2) K	
Wavelength	0.80000 Å	
Crystal system	Trigonal	
Space group	$P\,3_1$	
Unit cell dimensions	a = 29.430(4) Å	α = 90°.
	b = 29.430(4) Å	β = 90°.
	c = 13.520(3) Å	γ = 120°.
Volume	10141(4) $Å^3$	
Z	3	
Density (calculated)	1.234 Mg/m^3	
Absorption coefficient	0.221 mm^{-1}	
F(000)	3963	
Crystal size	0.100 × 0.100 × 0.050 mm^3	
Theta range for data collection	1.558 to 28.875°.	
Index ranges	$-35<=h<=35$, $-35<=k<=35$, $-16<=l<=16$	
Reflections collected	119096	
Independent reflections	24764 [R(int) = 0.0341]	
Completeness to theta = 28.685°	99.9 %	
Absorption correction	Semi-empirical from equivalents	
Max. and min. transmission	1.000 and 0.741	
Refinement method	Full-matrix least-squares on F^2	
Data / restraints / parameters	24764 / 3246 / 2770	
Goodness-of-fit on F^2	1.985	
Final R indices [I>2sigma(I)]	R_1 = 0.1209, wR_2 = 0.2711	
R indices (all data)	R_1 = 0.1498, wR_2 = 0.2924	
Absolute structure parameter	0.07(2)	
Extinction coefficient	n/a	
Largest diff. peak and hole	0.359 and –0.262 e.$Å^{-3}$	
